# Convergence and Reducibility as Transferability Filters in Biomimetic Design

**DOI:** 10.3390/jfb17060272

**Published:** 2026-06-01

**Authors:** Ozren Polašek

**Affiliations:** 1 Croatian Science Foundation, 10000 Zagreb, Croatia; ozren.polasek@hrzz.hr; 2 Department of Public Health, University of Split School of Medicine, 21000 Split, Croatia

**Keywords:** biomimetic design, convergent evolution, evolutionary trade-offs, transferability, bioactive peptides, comparative histology, phenotyping, evidence-based biomaterials

## Abstract

Biomimetic design is often justified by the claim that evolution has refined biological systems under severe selective pressure; however, this claim is incomplete. Evolution does not produce optimal solutions, but constrained trade-off resolutions. The translational question is therefore not whether a biological system performs the desired function, but whether the functional principle can survive separation from the system that produced it. Convergent evolution, where distantly related lineages independently arrive at similar solutions to the same functional problem, raises the probability that such solutions reflect physical or chemical constraints, which are stronger candidates for transfer into biomaterial design. Lineage-isolated solutions require a different test, namely whether the function reduces to a feature that can be reproduced outside the source organism. The argument is demonstrated through a convergence × reducibility matrix and an ex natura protocol from a biological phenomenon to a testable biomaterial claim. Biomimetics earns its place not as a universal design doctrine, but in those situations where evolutionary trade-off resolutions can survive translation into safe and manufacturable biomaterials.

## 1. Introduction

Biomaterial development operates under several conflicting demands, including biological function, mechanical adequacy, manufacturability, biocompatibility, regulatory acceptability and durable in vivo performance [1]. Bio-inspired design enters this process with the assumption that biological systems have already solved many of the relevant functional problems [2,3]. The intuitive force of this proposition rests on a single argument; evolution has acted across vast time and under considerable selective pressure, and biological solutions are therefore unlikely to be arbitrary [4].

The argument is sound, but limited. Evolution does not optimise for biomedical use [5,6,7]. It does not maximise device lifetime, regulatory predictability, manufacturing tolerance or cost; it produces locally viable compromises [8]. A biological mechanism may be remarkable precisely because it is inseparable from the metabolically active, developmentally patterned and immunologically regulated system that sustains it. Salamander limb regeneration [9], naked mole-rat resistance to neoplasia [10] and foetal scarless healing [11] are scientifically profound; however, they are not, by that fact alone, scaffold designs.

Existing reviews of biomimetics in biomaterials largely catalogue and illustrate translation through case studies [12,13,14]. A pre-translation test linking evolutionary recurrence, reducibility and comparator performance before engineering effort is committed remains underdeveloped. The dominant heuristic, which rests on biological resemblance alone, is a poor predictor. A coating that visually resembles a lotus leaf does not necessarily reproduce its wetting behaviour [15,16]. A scaffold that imitates the layered geometry of nacre does not necessarily reproduce its damage tolerance [17,18,19]. A device labelled “regenerative” does not necessarily recreate the cellular and immune conditions required for tissue restoration [20,21,22].

This review advances two main arguments. Firstly, evolution should be understood as a generator of survivable trade-off resolutions rather than as an optimisation engine, and this distinction has direct implications for which biological models are worth copying and which are not [23]. Secondly, convergent evolution, defined as the independent arrival at similar solutions in distantly related lineages [24,25], provides an additional operational filter for transferability by raising the prior probability that the solution reflects a physical or chemical attractor and not a developmental contingency. Convergent solutions are therefore more likely to reflect the deep or irreducible physical or chemical constraints that can be harnessed and re-used in engineering practice [26,27]. Narrower lineage-isolated solutions are less likely to be directly transferable, since they may have evolved in a more specific biological context.

The argument is developed as a recurring analytical lens rather than as a single-problem review. In addition, the practical output is an ex natura protocol that forces a biomimetic claim to specify the phenotype, source-target tissue relation, trade-off, transferable unit, manufactured embodiment and comparator evidence before the claim is treated as more than biological analogy (termed ex natura by analogy with ex vivo, denoting the staged movement from biological source to laboratory characterisation).

This is a narrative review and it does not aim to catalogue all biomimetic biomaterials. The scope is restricted to biomaterials and adjacent therapeutic material systems, including surfaces, structural composites for hard tissue, hydrogels and matrices, adhesives, drug delivery vehicles, antimicrobial interfaces and material-bound bioactive motifs. Other biomimetic domains, such as architecture, robotics and packaging, are outside the analytical frame of this review.

## 2. Biological Inspiration, Biomimetic Abstraction and Bioinformed Design

Three distinctions matter for the arguments that follow (Figure 1). Biological inspiration is the broad use of living systems as analogies and influences metaphor and rhetoric more than mechanism [28]. Biomimetic abstraction is the attempt to extract a functional principle, such as a surface geometry, a hierarchical architecture, a chemical motif or a dynamic feedback rule, from a biological system, and to re-express it in synthetic form [2,28,29]. Bioinformed design integrates biological data, environmental context and engineering constraint into design decisions, without necessarily committing to direct imitation [28].

This distinction matters because superficial resemblance is not the same as functional transfer. Biomimetics should therefore be judged by abstracted function and not by visual similarity to a biological referent [30]. For example, a dental composite that reproduces the cross-lamellar architecture of nacre is biomimetic in a defensible sense [18,19,31,32,33], while a coating that is merely iridescent is not.

The failure modes of biomimetic design follow from this distinction. Visual resemblance copies appearance without the underlying causal mechanism. Organismal copying attempts to transfer systemic traits without the regulatory system that makes them work. Only mechanistic abstraction, namely the identification of the relevant principle, its expression in a form independent of the original biological substrate and its testing against the engineering problem, can be said to earn the biomimetic label in any meaningful sense [2,26].

Organism-derived biomimetics infers principles from natural history, while laboratory evolution and in vitro selection differ in that selection occurs inside the design loop and is not inferred from observations of natural systems [4,34,35,36]. The two approaches are conceptually adjacent, since both rely on selection under constraint; however, they produce different epistemic objects.

## 3. Evolution as a Trade-Off Engine, Not an Optimisation Engine

The strongest theoretical basis for biomimetic biomaterial design is that biological systems embody constraint-tested resolutions of competing performance demands [3,5,27]. These problems are also biomaterial problems. Implant coatings must integrate with the surrounding tissue without provoking fibrosis [37]. Adhesives must grip wet and dynamic surfaces without cytotoxicity [38,39]. This structural similarity between biological and biomaterial problems is what makes biomimetics worth attempting in the first place.

The intuition that evolution produces optimal solutions is powerful, but biologically unsafe. Four mechanisms are particularly relevant for biomimetic transfer, not as textbook evolutionary decoration, but as direct reasons why biological form cannot be copied as if it were an engineering optimum (Supplementary Note S1) [5,6,40,41,42,43,44,45]. The consequence of this is not anti-biomimetic scepticism, but a more disciplined form of biomimicry. Evolution supplies constraint-tested compromises and not optimal blueprints. The transferable object is therefore never the organism as such, but the separable principle that can survive abstraction from it.

Consequently, biological singletons may be weak transfer candidates, since a trait found in a single species or developmental context is more likely to reflect contingency than constraint. Secondly, recurrent biological solutions are stronger candidates, since when the same functional problem produces structurally similar solutions in lineages that diverged hundreds of millions of years ago, such convergence can be taken as evidence that the solution reflects something deeper than historical accident [24,25]. Thirdly, the reducibility of the function to a defined feature matters separately, since even a singleton may transfer if the functional unit is small enough to be extracted from the organismal context in which it evolved [2,26,28].

## 4. Convergent Evolution as a Transferability Filter

Convergent evolution is the independent acquisition of similar traits in lineages that did not inherit them from a common ancestor. Camera-type eyes have evolved independently in vertebrates and cephalopods [46]. Echolocation evolved independently in bats and toothed whales, with parallel changes occurring at the same loci [47]. Crab-like body plans have arisen at least five times, an observation so recurrent that it has acquired its own designation, namely carcinisation [48]. Notably, functional convergence can also appear within a single organism, but it is not convergence in a cladistic sense (Supplementary Note S2).

### 4.1. Levels of Convergence

Not all convergence carries equal weight for biomaterial design [24,25]. Four degrees of convergence can be defined, each with different underlying mechanisms and implications for biomimetic development.

Functional convergence indicates that the same problem recurs across lineages and is solved in some way—organisms attach to substrates [49,50], resist fracture [18,19] and exclude microbes [51,52,53]—but the term does not specify how the solution is achieved. Functional convergence therefore establishes the problem as real and relevant, but not the solution as transferable.

Structural convergence indicates that similar architectures or geometries are repeatedly used to solve the recurrent problem, such as hierarchical fibrillar contacts for adhesion, stiff platelets in a compliant matrix for fracture resistance, or periodic micro- and nano-structures for fouling control [54]. This represents stronger evidence, since the constraint operates not only on the existence of a solution but also on its form.

Mechanistic convergence indicates that similar causal principles are repeatedly recruited across lineages, such as van der Waals interactions, crack deflection through interface sliding, or contact-mediated mechanical disruption of bacterial membranes [13,19,49,51,52]. This is the strongest evidence of transferability, since the mechanism itself is the object that engineering can reproduce.

Material convergence indicates recurrence at the level of chemistry or composition. It is often harder to interpret than mechanistic convergence, because recurring biological chemistry may reflect biosynthetic availability, phylogenetic inheritance, or cellular compatibility rather than performance alone [3,13,28].

### 4.2. Qualifications

Three qualifications are required at this point. The first is that convergence is not in itself a guarantee of transfer. Convergence raises the prior probability that a useful biomaterial principle is present; however, it does not determine downstream feasibility [2,27,28].

The second qualification is that the absence of convergence does not constitute a veto. Some lineage-specific solutions do transfer well, since the relevant function may be local in the sense that it reduces to a chemical motif, a surface protein or a defined domain structure, and may not depend on the rest of the organism in any substantive way. Mussel adhesive proteins are the canonical example of this situation [39,55,56]. The operative rule is therefore not that non-convergent solutions should be disregarded, but that convergent solutions deserve a different epistemic treatment, in that they come with stronger prior evidence that the underlying functional principle is real.

The third qualification concerns deep homology. Some apparent convergence partly reflects conserved developmental toolkits and not truly independent invention [57,58,59]. This does not nullify the transferability inference; however, it changes the meaning of that inference. The recurrence may signal a constrained developmental route rather than repeated de novo discovery. For biomaterial design, the target nevertheless remains the same, namely the transferable physics, chemistry, geometry or cue that can be reproduced without the organismal programme that originally produced it.

### 4.3. Convergence as the Worked Example

Hierarchical fibrillar dry adhesion has evolved independently in geckos and anoles, spiders, several insect groups and certain arboreal frogs [49,50]. The recurring solution is the subdivision of contact area into hierarchically arranged fine fibrils, which increases real contact area and exploits van der Waals interactions on dry and chemically diverse substrates [49]. The mechanism is the contact-splitting effect, in which adhesive force scales with the number of subdivisions, subject to geometrical and elastic constraints of the fibrillar tip [50]. The principle is reducible, since it is governed by a small number of parameters (fibril diameter, aspect ratio, tip geometry, modulus) that do not require an organismal network for their operation. Patterned elastomeric and polymer surfaces have reproduced the contact-splitting principle in manufacturable form, with chemistry-augmented variants extending function to wet substrates [39]. Performance gaps remain in durability under contamination and cycling, and matched comparisons against medical-grade adhesives are uneven [38]. Convergence identifies a constraint-tested principle, reducibility allows it to be specified and rebuilt, and the remaining engineering work concerns durability, biocompatibility and comparator performance rather than the principle itself.

Each convergence example translates into a defined tissue-engineering line of work. The recurrence of hierarchical biomineralisation in distantly related lineages [13] underwrites the active programme of nacre-mimetic and bone-mimetic composites for load-bearing scaffolds and dental restorations [3,17,60], where stiffness–toughness reconciliation is the limiting design problem [61]. The recurrence of cationic amphipathic antimicrobial peptides across kingdoms [62,63] underwrites peptide-functionalised hydrogels, wound dressings and antimicrobial coatings used in dermal, ocular, oral, gastrointestinal and orthopaedic applications [64,65,66]. In each case, convergence does not by itself certify the biomaterial; it identifies the underlying principle as a stronger transfer candidate, on which the subsequent reducibility, embodiment and comparator checks can then operate.

## 5. Convergence × Reducibility Matrix and Case Applications

Convergence and reducibility together generate four cells, the predicted transferability of which is summarised in Table 1; notably, the matrix is intended as a pre-translation heuristic rather than a formal scoring system. Reducibility is a property of the source biology and refers to the number of distinct components whose joint operation is required to generate the function of interest. A function governed by a single protein, a defined surface chemistry or a short sequence motif is highly reducible. A function that requires the coordinated operation of many components across cell types, tissue layers and developmental time is not. Related concerns have been raised under the headings of biomimetic abstraction [2,26,28], the problem of scale in organismal design [67] and the disappearing biological content of bio-inspired products [68].

The matrix is not a rigid taxonomy, but a coarse triage tool. The position of any given biological model within the matrix can shift as the underlying biology becomes better characterised, and as the relevant functional unit is identified at progressively finer resolution.

Lipid restoration at depleted mucosal and serosal interfaces represents an interesting intermediate case. Pulmonary surfactant, meibomian lipid layers, the stratum corneum and joint surface lipids reduce surface tension, modulate friction, exclude pathogens and gate transport across functionally distant tissues [81,82,83,84,85]. Lipids are biochemically universal, so their recurrence at biological interfaces is not by itself evidence of independent selection on the same problem. The recurring element is therefore not the chemistry but the system architecture.

The biomaterial response to lipid loss at these interfaces is established in some clinical settings and emerging in others. Exogenous pulmonary surfactant preparations, derived from animal lungs or synthesised from defined components, are an established treatment for neonatal respiratory distress syndrome [86,87,88]. Lipid-based eye drops and meibomian lipid replacement therapies are an active area of dry eye disease management; however, the comparative evidence base across formulations is uneven [89]. Topical lipid restoration in dermatology, particularly with ceramide, cholesterol and free fatty acid preparations, has a developed evidence base in atopic dermatitis and other barrier-deficient skin states [85,90].

Nature-derived medicinal peptides occupy the opposite pole from organismal imitation. They are often phylogenetically narrow yet highly transferable, since the functional unit is small, sequence-defined, synthesisable, manufacturable and testable. Antimicrobial peptides from host defence systems, venom-derived peptides, marine peptides, bacterial lipopeptides, glycopeptides and ECM-derived adhesive or signalling sequences have already influenced medicinal chemistry, antimicrobial therapy, wound healing, drug delivery and the development of functionalised biomaterials [62,72,73,74,75,76,77,91,92].

The relevant trade-off here differs from the trade-offs encountered in structural biomimetics [23,93]. Peptides negotiate a different set of competing demands, namely potency against toxicity, membrane activity against selectivity, stability against clearance, immunomodulation against inflammation, and binding affinity against off-target effects [73,75]. Their biomimetic value lies in reducibility, in that a peptide can often be separated from the source organism and synthesised, modified, immobilised, cyclised, conjugated or embedded in a hydrogel [75,78], without the need to carry the animal or microbial system that originally produced it.

The convergence signal is, however, mixed. Broad antimicrobial peptide strategies recur across kingdoms, since membranes, charge, amphipathicity and innate defence impose recurring constraints [62,94]. The individual sequences, however, are often lineage-specific, signalling that natural peptides are powerful starting points, not finished medicines [73,75]. The translation depends on sequence–function mapping, structure–activity relationships, protease stability, delivery, immunogenicity, toxicity and comparative efficacy [73,75] against existing antibiotics, antiseptics, growth factors and wound-care materials.

For biomimetic design, peptide-bearing hydrogels, antimicrobial coatings, wound dressings and tissue-contact surfaces are particularly important, since they combine local molecular function with material-level control over release, presentation and degradation [78,79,95]. The claim should however remain sharp; nature-derived peptides are not persuasive simply because they are natural, but only when their local mechanism survives synthesis, engineering, delivery and comparative testing.

### Mapping Transferable Principles onto Biomaterial Classes

**Figure 2:**
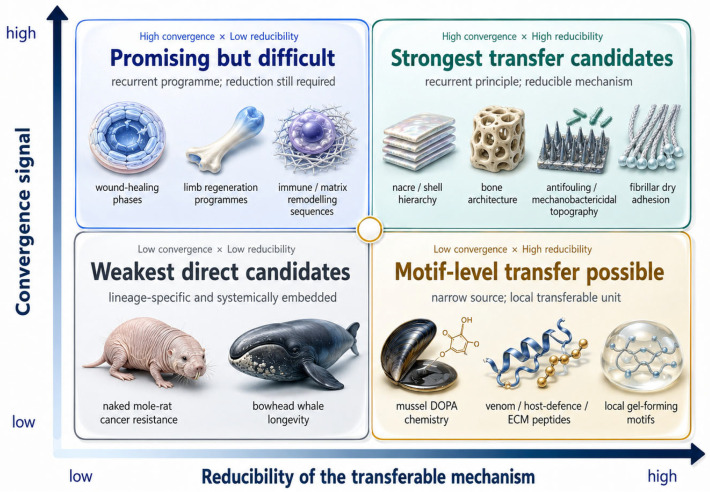
Convergence × reducibility matrix for biomimetic biomaterial transfer. Biological recurrence alone does not establish translational value, as candidate materials become stronger biomimetic targets when a convergent biological solution can also be reduced to a transferable mechanism. Highly convergent but mechanistically entangled systems provide strong biological signal but weak engineering objects, whereas clean but weakly recurrent systems may be useful opportunistic targets rather than priority exemplars. The strongest candidates occupy the transfer-priority zone, where recurrence supports biological relevance and reducibility permits abstraction, embodiment, and comparator-based validation.

Convergence and reducibility identify which biological principles are worth pursuing (Figure 2), but a principle is not yet a biomaterial development scheme. It becomes one only when it is realised in a specific material class and each class imposes its own manufacturing and regulatory constraints. Table 2 maps the high-convergence, high-reducibility examples discussed above onto the classes in which they are typically realised, together with the principal constraint that governs scale-up in each case.

## 6. Ex Natura Protocol: From Biological Phenomenon to Testable Biomaterial Claim

A biomimetic claim requires a protocol and not a metaphor. The ex natura route should be treated as a staged evidentiary pipeline, namely one in which only what can be named, measured, rebuilt and beaten against a comparator is extracted from nature. The protocol that follows is deliberately severe, since weak biomimetics typically fails before fabrication, in that it never proves that the copied feature is in fact the causal feature [2,28].

The first step is to define the biological phenotype quantitatively. The phenotype must be measured before it is admired [111], in terms such as adhesion force, fatigue resistance, bacterial kill, friction coefficient, diffusion profile, elastic modulus, wound-closure kinetics, inflammatory profile or cell-state shift. Terms such as “regenerative,” “natural,” “ECM-like” and “bioactive” are not, in themselves, phenotypes.

The second step is to perform comparative histology and anatomical mapping. The source and target tissues must be compared in terms of architecture, cell composition, matrix organisation, vascularity, innervation, immune exposure, mechanical loading, developmental stage and repair mode [111]. Without this step, a biomimetic gel or scaffold may copy the wrong tissue feature at the wrong biological scale.

The third step is to locate the trade-off. The biological system must be understood as solving a conflict, such as stiffness versus toughness, adhesion versus release, antimicrobial potency versus cytocompatibility, permeability versus barrier function, biological richness versus control, or degradation versus persistence. If no such trade-off can be named, no evolutionary argument has in fact been made [5,27].

The fourth step is to map convergence and reducibility. The protocol must ask whether the solution is recurrent across lineages and whether the functional unit reduces to a defined feature that can be extracted from its biological context [2,15,25,111].

The fifth step is to identify the transferable unit. The unit may take the form of a geometry, a hierarchy, a chemistry, a sequence, a ligand density, a viscoelastic relaxation, a degradation kinetic or a feedback rule; however, it cannot be the animal, the tissue, the phenotype name or the aesthetic resemblance [2,28].

The sixth step is to rebuild the unit in a manufacturable material. Synthesis, scale, sterilisation, storage, batch variation, degradation products, immune residue, regulatory classification and cost all enter at this stage [20,37]. The transition from a validated principle to a product candidate is governed by manufacturing and regulatory realities that are largely absent from the biological source. Preparation for application for human use, such as terminal sterilisation, must not destroy the transferred function. This may be a minor concern for an inert structural geometry but very relevant for peptides, lipids and catechol chemistries. Batch-to-batch consistency favours defined, synthesisable units over organism-derived extracts with intrinsic lot variability. Regulatory classification is determined by the embodiment rather than by the biology: a bare structural scaffold, a peptide-eluting scaffold and a growth factor-loaded scaffold that all mimic the same biological principle fall into device, combination-product and drug pathways respectively, each carrying a different evidentiary burden. Finally, cost scales with the embodiment—a principle that reduces to a manufacturable geometry or a short synthesisable sequence amortises far better than one that remains dependent on a biological source. A principle that cannot survive these constraints is not a product candidate, however sound the underlying biology.

The seventh step is to phenotype the engineered material and perform a substantial evidence-based comparative analysis. Better phenotyping is not optional. The material must be tested for the phenotype it claims to transfer, using quantitative imaging, histology, mechanics, molecular readouts, cell-state analysis, microbial assays and longitudinal stability assessment where relevant. For gels and biogels, this means measuring not only cell survival or histological filling, but also phenotype maintenance, matrix remodelling, degradation, immune response and mechanical integration. The comparator cannot be a blank plastic surface, an untreated wound or an obsolete scaffold in those situations where a better alternative exists. The proper question is whether the ex natura design outperforms the best available conventional, synthetic, semi-synthetic or clinically accepted material on the outcome that is supposed to justify the biomimetic claim.

**Figure 3:**
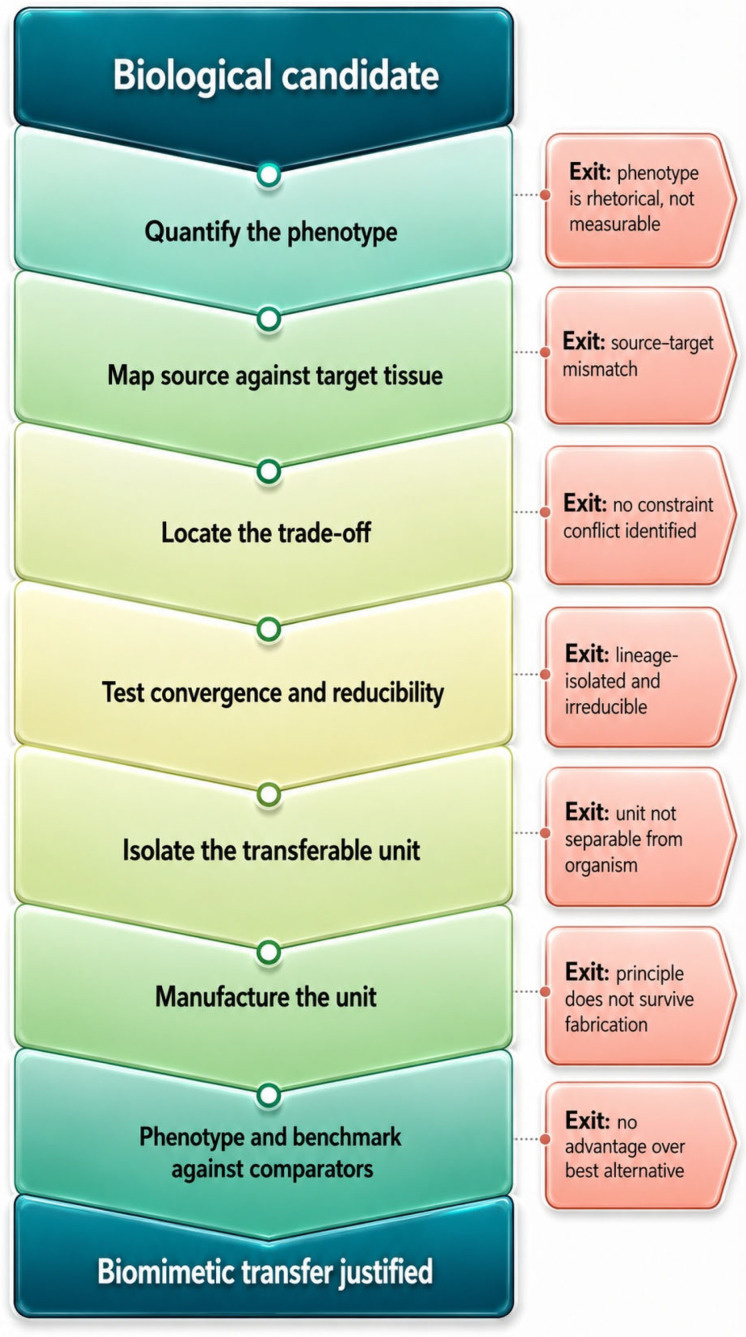
Ex natura protocol for converting a biological candidate into a justified biomimetic transfer claim.

The assumptions behind transfer therefore become explicit through this protocol. The source phenotype must be correctly measured. The relevant biological scale must be identified. The mechanism must be reducible to a defined feature. The target problem must share the same constraint structure. The engineered embodiment must preserve the causal principle. The final product must outperform serious alternatives. If any of these assumptions fail, the resulting claim may still be scientifically interesting; however, it can no longer be considered a biomimetic transfer claim (Figure 3).

## 7. Low-Transferability Biomaterial Targets

Three frequently invoked biological models occupy the low-convergence and low-reducibility cell of Table 1. Low transferability does not imply low biological importance. It means that the phenotype cannot be directly abstracted into a material principle without first identifying a smaller transferable unit, and validating that unit through comparative histology and explicit phenotyping.

The first case is salamander regeneration, phylogenetically restricted and not apparently convergent across vertebrates [9]. The relevant function is systemic and not reducible to a defined feature; the transfer mechanism is likely a regeneration-associated cue. The second case is cancer resistance and longevity. Naked mole-rat cancer resistance and bowhead whale longevity are striking phenotypes in long-lived mammals, but their mechanisms are lineage-specific rather than a single convergent material principle [10,80,112]. Their potential biomaterial relevance must therefore be identified at the molecular level. High-molecular-weight hyaluronic acid in naked mole-rat tissues, for example, is a candidate local motif whose rheological and signalling properties may transfer to soft-tissue applications, even if the organismal phenotype itself does not transfer [10]. The third is foetal scarless healing [11,113,114,115], a regulated developmental wound programme rather than a single material property; transferability here depends on a transitional developmental context that is essentially unreplicable at later developmental stages. These phenotypes can inspire biomimetic discovery efforts, especially about specific cytokine profiles, matrix compositions or macrophage-polarisation cues [116,117], but the systemic phenotype does not by itself specify a directly usable scaffold.

The pattern across these three cases points to a positive heuristic rather than a dead end. A systemic, lineage-restricted phenotype is not a transfer candidate, but it is a place to look for one. The operative move is to relocate the claim from the organism to the smallest defined unit that can be named, measured, reduced, and rebuilt, at which point the unit re-enters the convergence × reducibility matrix as an ordinary candidate. The biomaterial value of these phenotypes therefore lies at a smaller scale, in molecular discovery; it is not lost but relocated to a scale at which the ex natura protocol can act on it.

## 8. Research Agenda

The first priority is matched comparative analysis of biomimetic and conventional biomaterials by functional outcome (Supplementary Note S3). The biomimetic literature is rich in case studies but poor in matched comparisons [3,13,14]. Patent-based, preclinical, clinical trial-based and systematic review-level comparisons of biomimetic, synthetic, semi-synthetic and standard-care materials for the same indication would test whether bio-inspired origin predicts performance in any systematic way. The analysis should be stratified by transferability class, namely convergent and reducible cases, low-convergence and reducible cases, gel and matrix systems, and systemic phenotype claims.

Second, explicit phylogenetic and comparative histological mapping of biomimetic sources is required [24,25,111]. The current literature is fragmented and tends to treat biological models on a case-by-case basis. A systematic map should record not only whether a transferred principle is convergent in some sense, but also at which level it is convergent—namely functional, structural, mechanistic or material—and whether the source and target tissues are in fact histologically comparable at the relevant scale.

A third priority is improved phenotyping standards. The lack of agreed and reliable measurement protocols for the phenotypes that biomimetic designs claim to recreate is one of the main reasons that comparative claims remain weak [20,37]. A consensus on which phenotypes must be measured, and how, would substantially raise the evidentiary bar.

Fourth, translation-failure analysis is needed. Failed biomimetic translations are arguably more informative than successful ones, since they reveal where the principle actually breaks down, whether in abstraction, fabrication, biocompatibility, durability, regulation, delivery, phenotype fidelity or comparator performance. The failure-case literature is, however, comparatively sparse [67,68]. This bias is scientifically costly.

## 9. Conclusions

Biomimetic design is most defensible in those situations where evolution has revealed a physical or chemical constraint that engineering can act on. Convergent evolution provides an operational filter for predicting where such constraints exist, while reducibility determines whether the constraint can be specified at a scale at which engineering can intervene. The proper question for biomimetic development is therefore not whether biological inspiration is superior to synthetic engineering as such. The real question, then, is whether evolutionary trade-off resolutions can survive translation into safe, manufacturable biomaterials compatible with regulatory norms, and whether the resulting materials outperform the alternatives after convergence, reducibility, comparative histology, phenotyping and matched comparative evidence have done their work.

## Figures and Tables

**Figure 1:**
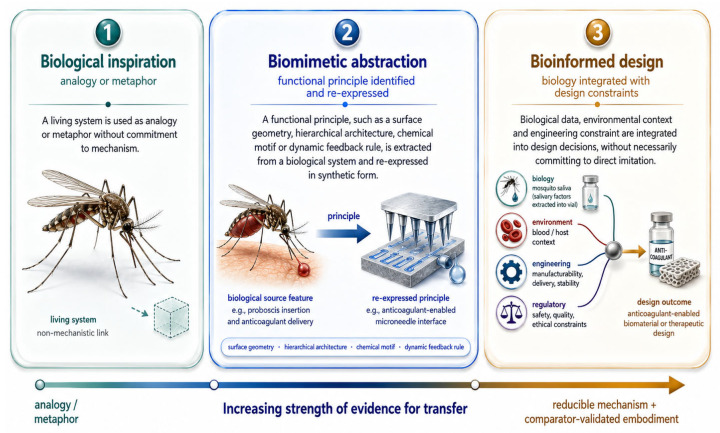
Three modes of biological involvement in materials design, ordered by the strength of evidence required to support a transfer claim. Biological inspiration uses living systems as analogy or metaphor and carries little mechanistic burden. Biomimetic abstraction requires identification of a transferable functional principle and its re-expression in synthetic form. Bioinformed design integrates biological mechanism, environmental context, engineering constraints, and regulatory requirements into a comparator-validatable design pathway. The evidentiary burden therefore increases from left to right, from suggestive analogy to reducible mechanism and embodied performance.

**Table 1:** Convergence × reducibility matrix for biomimetic biomaterial transferability.

	High Reducibility (Function Reduces to Chemistry, Physics, Geometry, Surface or Domain)	Low Reducibility (Function is Systemic, Regulated and Developmentally Embedded)
High convergence	*Strongest transfer candidates*. Examples include hierarchical biomineralisation in nacre and bone [18,19,31,32,67,69,70], antifouling and mechanobactericidal topographies [51,52], and dry adhesion via fibrillar contacts [39,49,50].	*Promising but difficult*. Recurrent biological programmes may reveal conserved mechanisms; however, direct material transfer requires reduction to defined cues, domains, gradients or feedback rules. Examples include conserved wound-healing phases [21], limb regeneration [9,71], recurring innate immune patterning and matrix-remodelling sequences [20,22].
Low convergence	*Motif-level transfer is possible*. Examples include mussel DOPA chemistry [39,55,56]; selected venom-, host-defence- or ECM-derived peptides [62,72,73,74,75,76,77]; and local gel-forming motifs [78,79]. The burden of proof falls on the reducibility, dose–response, stability and deliverability of the functional unit.	*Weakest direct candidates*. Examples include naked mole-rat cancer resistance [10] and bowhead whale longevity [80].

**Table 2:** Transferable principles mapped onto biomaterial classes, with the principal manufacturing constraint governing scale-up.

Transferable Principle	Biomaterial Class	Representative Embodiment	Principal Manufacturability/Scale-Up Constraint
Hierarchical biomineralisation (nacre, bone) [13]	Structural composites; hard-tissue scaffolds	Nacre-mimetic mineral–polymer composites; mineralised scaffolds [3,17]	Ordered “brick-and-mortar” architecture is difficult to reproduce at bulk thickness and scale; layer-assembly and freeze-casting routes are slow and size-limited [96,97]
Mechanobactericidal/antifouling topography [52]	Surfaces and coatings	Nanostructured implant surfaces; contact-killing films [98,99]	Nanoscale features must survive sterilisation, handling and tissue contact; pattern fidelity over large or curved areas is hard; durability under fouling is unproven [100,101]
Fibrillar dry adhesion (contact splitting) [102,103]	Surfaces; adhesives	Patterned elastomeric/polymer adhesive films [104,105]	Moulding is scalable, but contamination tolerance, cyclic durability and wet-substrate performance remain unsolved [106]
Mussel-derived catechol (DOPA) chemistry [56,107,108]	Adhesives; coatings; hydrogel crosslinkers	Catechol-functionalised adhesives and surface coatings [56,108]	Catechol oxidation must be controlled for shelf life and batch consistency; wet-cure kinetics are formulation-sensitive [109,110]
ECM- and host defence-derived peptide motifs [62,63,72]	Hydrogels; coatings; wound dressings; particulate carriers	Peptide-functionalised hydrogels; antimicrobial coatings [64,65,95]	Solid-phase synthesis is scalable for short sequences, but cost rises steeply with length; protease stability and immunogenicity gate translation; sterilisation can degrade activity [66]

## Data Availability

No new data were created or analyzed in this study. Data sharing is not applicable to this article.

## Supplementary Materials

The following supporting information can be downloaded at: https://www.mdpi.com/article/10.3390/jfb17060272/s1, Supplementary Notes S1. Evolutionary processes relevant for biomimetic development; Notes S2. Single-organism convergence; Notes S3. Biomimetic vs synthetic development.
