# Supplementary material for: Convergence and Reducibility as Transferability Filters in Biomimetic Design
Source: J Funct Biomater. 2026 Jun 1;17(6):272. doi: 10.3390/jfb17060272 (PMC13301601; doi:10.3390/jfb17060272)
Supplement: Supplementary file 1 [file jfb-17-00272-s001.zip › jfb-4326446-supplementary.pdf]

Convergence and reducibility as transferability filters in biomimetic design
Supplementary material
Ozren Polašek

Supplementary Note S1. *Evolutionary processes relevant for biomimetic development*

Four evolutionary mechanisms relevant for biomimetics include genetic drift, historical contingency, exaptation and developmental & pleiotropic constraint.

Genetic drift means that molecular or microstructural detail may be functionally incidental. Neutral or near-neutral variants can become fixed by chance, so that precise copying may reproduce historical residue rather than functional performance [40]. Drift matters because copied molecular detail may turn out to be functionally incidental, in which case the amino acid composition of a structural protein may reflect neutral fixation and not performance, and reproducing it precisely buys nothing in engineering terms.

Historical contingency means that organisms build from inherited structures and not from a blank design space, and a given architecture may therefore persist because development permits it, not because it is globally optimal for the present function [6,39]. Contingency matters because copied architecture may persist for reasons of developmental history rather than performance, and a scaffold that reproduces the precise geometry of trabecular bone may inherit features that exist only because bone is metabolically active and continuously remodelled.

Exaptation means that the present function of a structure may be a later co-option of a feature originally shaped for another role [41], and the biomimetic designer who extracts only the visible function risks missing the ancestral constraints still embedded in the mechanism. Exaptation matters because the present function of a structure may not reveal its original design logic, and a biological mechanism extracted for one biomedical purpose may carry residual constraints from an unrelated ancestral role.

Developmental and pleiotropic constraint means that a trait is often viable only inside a network of linked effects [42-44], and the removal of a component from that network can destroy the very performance that made the trait attractive in the first place. Pleiotropy matters because an isolated material component may fail once it is removed from the organismal network that made it viable, and the decoupling of function from substrate then becomes the central translation problem.

Supplementary Note S2. *Single-organism convergence*

The convergence in strict sense is cross-lineage: similar solutions appearing in groups that did not inherit them from a common ancestor. A related pattern occurs within a single organism, where anatomically and developmentally distinct systems independently arrive at the same solution to the same physical problem. This is not convergence in the strict cladistic sense, since the sites in question share a genome and often a developmental toolkit. For the transferability question, however, within-organism recurrence carries a signal that is both analogous to cross-lineage convergence and, in one respect, cleaner.

It is analogous because the explanatory logic is unchanged. When systems that serve different organs, arise from different embryological territories and are under different regulatory control nonetheless settle on the same mechanism, the most parsimonious explanation is again the physics or chemistry of the shared problem rather than historical accident. The deep-homology qualification applies here with more force, since the recurring solution may reflect a developmental toolkit redeployed at several sites rather than independent invention. As before, this changes the meaning of the inference without nullifying it: whether the recurrence is independent or redeployed, the transferable object remains the physics, chemistry or geometry that can be reproduced outside the organism.

It is cleaner in a methodological sense. Cross-lineage comparison forces a histological match between source and target tissues across species, which is the step at which many biomimetic claims

quietly fail. Within-organism convergence removes that confound: the two systems being compared sit in the same body, so the comparative histology required by Step 2 of the ex natura protocol becomes a within-organism comparison with the species-matching problem eliminated.

Supplementary Note S3. *Biomimetic vs synthetic development*

The opposition between biomimetic and synthetic biomaterials is largely false. Most biomimetic biomaterials are in fact synthetic in their execution [2,3,28], and the meaningful contrast is therefore between bio-inspired and conventional design pathways, and not between biomimetic and synthetic ones. Bio-inspired pathways are stronger in situations where the problem is multi-objective, the design space is poorly explored, or the conventional paradigm has converged on a local optimum that is unsatisfactory in some respect. A mature biomaterial development process will therefore often begin bio-inspired and finish synthetic, in that biology provides the candidate principle while engineering provides safe and manufacturable product.

This reframing changes the comparative claim. Biomimetic design is not categorically superior to conventional design. It is a search strategy combined with a translation filter. It earns its place in those situations in which the biological model identifies a principle that conventional engineering would not have proposed, in which that principle survives abstraction and manufacture, and in which the resulting material beats a serious comparator. Convergence and reducibility estimate, before engineering investment is committed, whether a principle is likely to survive translation; matched comparative analysis decides whether it actually did.

References correspond to the reference list from the main document.
